# Increased reoperation rates after meniscus repair compared to arthroscopic partial meniscectomy: Data from a comprehensive clinical cohort with up to 10 years follow‐up

**DOI:** 10.1002/ksa.12791

**Published:** 2025-07-21

**Authors:** Fredrik Boric‐Persson, Aleksandra Turkiewicz, Martin Englund, Paul Neuman

**Affiliations:** ^1^ Department of Orthopaedics, Skåne University Hospital Lund University Malmoe Sweden; ^2^ Department of Clinical Sciences Lund, Orthopaedics, Clinical Epidemiology Unit, Faculty of Medicine Lund University Lund Sweden

**Keywords:** arthroscopy, meniscus repair, meniscectomy, knee surgery, reoperation

## Abstract

**Purpose:**

To investigate the rates of knee reoperation and medical complications after meniscal repair versus partial meniscectomy (APM) up to 10 years after surgery.

**Methods:**

All patients ≥ 15 years old operated for a meniscal tear with meniscus repair or partial meniscectomy at Scania University Hospital were included, between year 2010 and 2014. Information was retrieved from patient records until the year 2020. Rates of any reoperation, reoperation in same meniscus and medical complications were estimated. Differences were also estimated in the three outcomes in a subgroup aged 15–40 years using flexible parametric survival models adjusted for age, sex, knee laterality, tear type, medial/lateral, anterior cruciate ligament (ACL) status, osteoarthritis, body mass, height and smoking.

**Results:**

Records identified 2098 patients (395 undergoing meniscal repair and 1703 partial meniscectomy) with 540 reoperations in 430 patients. The incidence rate of reoperation was 32/1000 person‐years (95% confidence interval [CI] 29–35) and of reoperation in the same meniscus 19/1000 person‐years (95% CI 17–21). There were 2.1% postoperative complications. In the age group 15‐40 years, with only bucket‐handle, longitudinal and horizontal tears, 341 patients had meniscal repair and 361 partial meniscectomy. The incidence rate of any reoperation was 105 (95% CI 90–122) per 1000 person‐years in the meniscal repair group and 24 (95% CI 18–31) in the partial meniscectomy group. The adjusted hazard ratio of any reoperation comparing meniscal repair with partial meniscectomy was 4.3 (95% CI 3.1–6.0) and of reoperations in the same meniscus 17 (95% CI 9–31). 3.3% patients had postoperative complications (15 after meniscal repair and 14 after partial meniscectomy).

**Conclusions:**

The risk of any knee reoperation after meniscal repair had a four‐fold increase compared with partial meniscectomy, and for same meniscus reoperations about 17‐fold. The rate of medical postoperative complications was low.

**Level of Evidence:**

Level III.

**Abbreviations**
ACL: anterior cruciate ligament
ACLR: anterior cruciate ligament reconstruction
APM: arthroscopic partial meniscectomy
ASA: American Society of Anaesthesiologists
BMI: body mass index
CI: confidence interval
HR: hazard ratio
ICD10: International Classification of Diseases, version 10
ICRS: International Cartilage Repair Society
KOOS: Knee injury and Osteoarthritis Outcome Score
NCSP: Nordic Medico‐Statistical Committee
OA: osteoarthritis
SD: standard deviation
VTE: venous thrombus embolism

## INTRODUCTION

Arthroscopic partial meniscectomy (APM) is the most common type of knee surgery with an estimated numbers of procedures close to a million per year in the United States [12]. In recent years, the trend has been to treat meniscus injuries with repair, instead of partial meniscectomy when possible [1, 11, 23, 24]. In Sweden (population 10.4 million, 2021), there were more than 8000 meniscus surgeries in 2012, with an incidence rate of arthroscopic knee surgery for acute traumatic meniscus tears of 13.7 per 100,000 person years [18]. Meniscal repair for acute tears could lead to a lower risk of knee osteoarthritis (OA) as compared to partial meniscectomy [20]. However, repair of the meniscus requires more time in the operating room than partial meniscectomy and longer rehabilitation period with activity restrictions, longer absence from work and slower return to sports [3, 15, 39]. Still, meniscal repair is reported to be cost‐effective compared to partial meniscectomy in the treatment of traumatic meniscal injuries, but not in the presence of knee OA [8].

The risk of meniscus repair failure is reported to be around 15% (ranging from 0% to 44%) [4, 22, 26, 27, 29, 32, 33, 38], whereas studies on partial meniscectomy often reports lower frequencies of complications and number of reoperations [10, 28, 31]. Since the studies of Stein et al. in 2010 and Paxton et al. in 2011, reports of a large cohort comparing partial meniscectomy versus meniscus repair are limited [21, 25, 31, 37]. There is a lack of recent studies comparing partial meniscectomy and meniscus repair in a large patient material without patient selection, with clear definitions of reoperations and treatment failure outcomes.

Thus, the aim of this study was to investigate the risk of reoperation of the ipsilateral knee for all causes and reoperation of the same meniscus (true failure) after meniscal repair and partial meniscectomy, as well as other medical complications in a well‐defined large clinical cohort with a follow‐up of 5–10 years. The second aim was to compare meniscal repair and partial meniscectomy regarding risk of reoperation of the ipsilateral knee for all causes, reoperation of the same meniscus and other medical complications in a subgroup of patients aged 15–40 years with tears potentially suitable for meniscal repair. The hypothesis was that meniscus repair is associated with increased rates of reoperations and complications compared to partial meniscectomy.

## MATERIALS AND METHODS

The study was approved by the Regional Lund University Ethics Committee (IRB 2016‐873/2017‐02‐07 and IRB 2019‐02850/20191121) and it adheres to the rules of the Helsinki Declaration of 1975, as revised in 2000.

A cohort study was performed using prospectively ascertained healthcare data from the most southern region in Sweden, Scania (population 1.3 million). The OrtReg database was used and it has been developed by orthopaedic surgeons in Scania. All orthopaedic operations performed by the public healthcare in the region are registered in it and linkable by the patient's unique personal identification number providing information of birthdate, sex, current residential address and death of the patients. It contains diagnostic codes according to the International Classification of Diseases, version 10 (ICD‐10) and surgical procedure codes according to the Nordic Medico‐Statistical Committee (NCSP) as well as a wide range of data about the procedures performed and tissue observations, which is filled in by the surgeon after each surgery.

To record postoperative complications and to supplement patient characteristics the Orbit system and the Melior patient medical records were reviewed. Orbit is the regional surgical planning system containing all intraoperative medical data from all surgeries in the region. Melior is a digital medical record and contain diagnostic codes from any visit to a public hospital in the region. From Melior and Orbit we retrieved preoperative data comprising age, gender, smoking status, weight, height, medications administered intraoperatively and presence of other diseases in the knee joint. Journal text and registered diagnosis were screened for adverse events by the junior author (FBP). In cases where there was any doubt whether to include it as a knee surgery related complication or not, it was also reviewed by the senior author (PN).

### Definition of reoperations and complications

The primary outcomes of interest were reoperation for all causes and reoperation in the same meniscus. The second outcome of interest was complications. Any new surgery done in the ipsilateral knee during the follow‐up was considered a reoperation. A reoperation in the same meniscus (i.e., meniscus surgery failure) was defined as new meniscal surgery in the same meniscus (medial vs. lateral) during follow‐up. Total knee replacement was considered a reoperation in the same meniscus for both medial and lateral menisci. A complication was any serious adverse medical event found in the medical records up to 2 years postoperatively that maybe could be directly linked to the knee surgery. This longer period was chosen to also capture slowly evolving complications like arthrofibrosis [6, 35]. Local pain, swelling, hemarthrosis not in need of treatment, and local loss of skin sensation, were anticipated and not regarded as serious medical complications.

### Cohort inclusion and exclusion criteria

All patients matching the following inclusion criteria were identified: (a) NCSP‐code of NGD11 (partial meniscectomy) and/or NGD21 (meniscus suture) stated in the surgical report; (b) hospital of surgery: Malmoe, Lund or Trelleborg; (c) year of surgery: 2010–2014; (d) age 15 years or older at time of surgery; and (e) resident in Scania. Each person could only be included once, if bilateral knee surgery, the first surgery in the records was selected.

**Figure 1:**
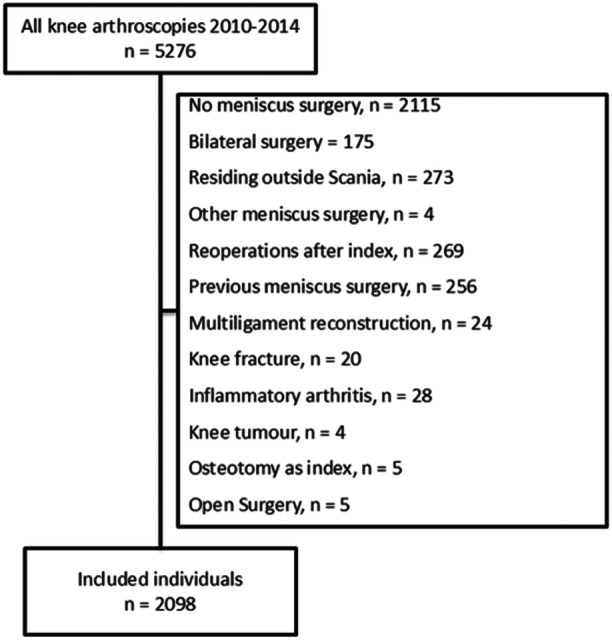
Flowchart of the study.

Patients were excluded according to the schedule presented in Figure 1. Baseline group characteristics are displayed in Table 1.

**Table 1:** Baseline characteristics of the cohort.

	All patients^a^	Subgroup 15–40 years^b^		
		All	APM	Meniscus repair
*N*	2098	702	361	341
Age, years, mean (SD)	39.0 (14.7)	26.2 (7.3)	27.9 (7.1)	24.4 (7.1)
Female, *N* (%)	701 (33.4)	223 (31.8)	90 (24.9)	133 (39.0)
Length, cm, mean (SD)*	176.3 (9.0)	177.1 (9.1)	178.2 (9.1)	176.0 (8.9)
Weight, kg, mean (SD)*	82.2 (15.7)	78.7 (14.1)	80.6 (14.0)	76.7 (14.0)
BMI, mean (SD)*	26.4 (4.2)	25.0 (3.5)	25.3 (3.5)	24.7 (3.6)
Smoker, *N* (%)*	290 (14.3)	103 (14.9)	63 (17.8)	40 (11.9)
Right knee, *N* (%)	1080 (51.5)	377 (53.7)	196 (54.3)	181 (53.1)
Knee OA, *N* (%)	754 (35.9)	76 (10.8)	44 (12.2)	32 (9.3)
ASA grade*				
1, *N* (%)	1390 (73.0)	556 (85.0)	279 (84.0)	277 (86.0)
2, *N* (%)	495 (26.0)	97 (14.8)	53 (16.0)	44 (13.7)
3, *N* (%)	20 (1.0)	1 (0.2)	0 (0)	1 (0.3)

Abbreviations: APM, arthroscopic partial meniscectomy; ASA, American Society of Anaesthesiologists; BMI, body mass index; OA, osteoarthritis; SD, standard deviation.

^a^ Missing data: length *n* = 66 (3.1%), BMI *n* = 66 (3.1%), weight *n* = 62 (3.0%), smoker *n* = 77 (3.7%) and ASA grade *n* = 193 (9.2%).

^b^ Missing data group 15–40: length: APM *n* = 10 (2.8%) meniscus repair *n* = 6(1.8%), weight APM *n* = 8 (2.2%) meniscus repair *n* = 6(1.8%), BMI, APM *n* = 10 (2.8%) meniscus repair *n* = 6 (1.8%), smoker APM *n* = 8 (2.2%) meniscus repair *n* = 4(1.2%), ASA grade: APM *n* = 29 (8.0%) meniscus repair *n* = 19 (5.6%).

### Subgroup of patients 15–40 years old

**Figure 2:**
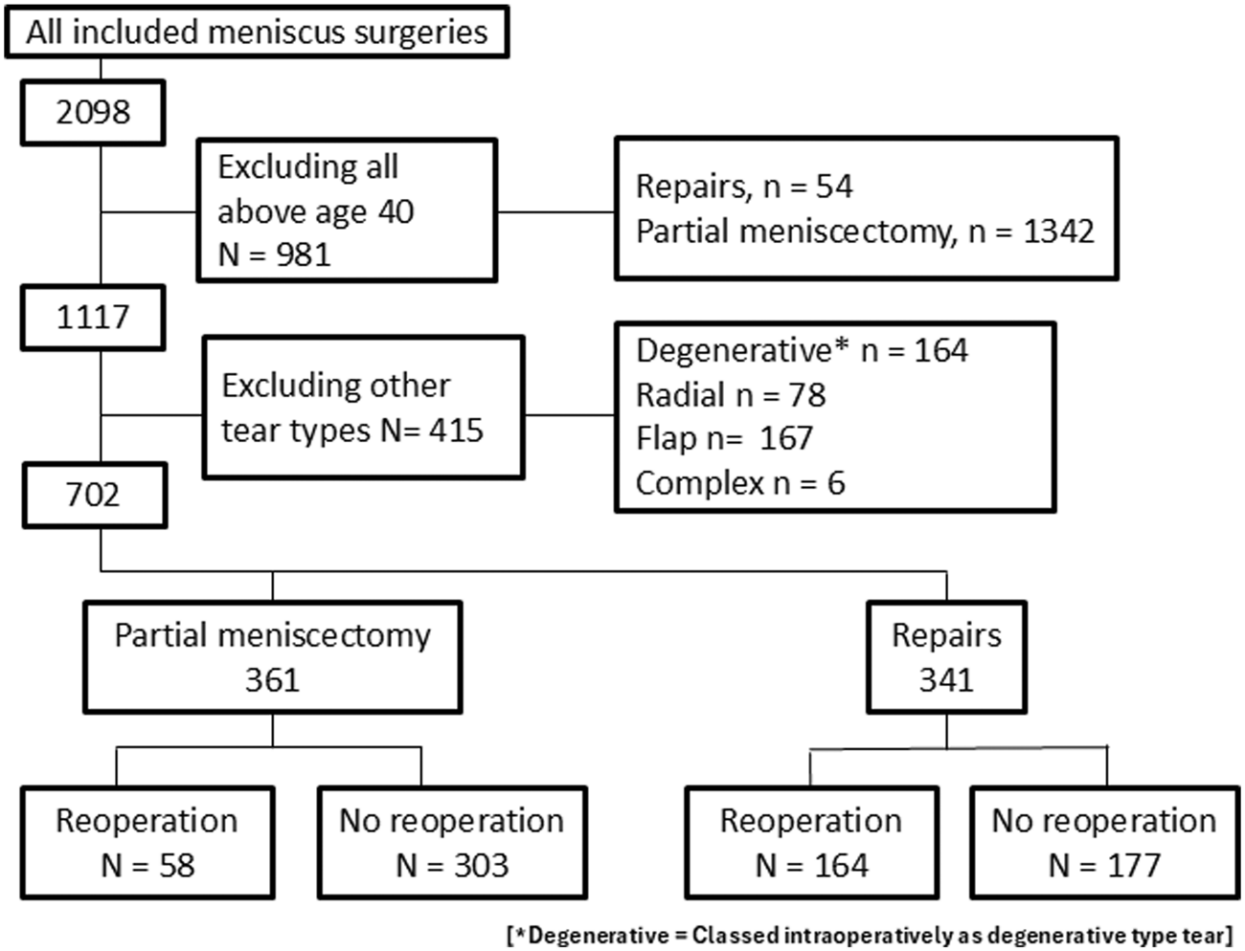
Flowchart for subgroup 15–40 years old.

For the second aim, to compare reoperations rates between meniscal repair and partial meniscectomy, a more homogenous cohort of patients between 15 and 40 years of age was created, depicted in Figure 2, to exclude most degenerative meniscal injuries, accounting for that the meniscus quality and rate of meniscal repair tend to decrease with age. It also included only types of meniscal tears typically amendable for repair, namely tears classified as bucket‐handle, longitudinal or horizontal tears, as these could theoretically be eligible for either partial meniscectomy or meniscal repair [30].

Knee stability was classified into three groups: (1) Stable knees at preoperative examination, (2) unstable knees, ACL‐tears or previous ACLR with unstable knee at preoperative examination and (3) ACLR at index surgery.

### Data collection

Intra‐operative data were collected regarding type and locations of meniscus tear, type and location of surgery, all specified as medial or lateral, status of cartilage and ligaments and other surgery performed in the index knee. In cases of concurrent meniscal repair and partial meniscectomy, the surgery was considered as meniscal repair. Knee OA was recorded from the patients' preoperative medical records or intra‐operative diagnosis by the surgeon. Knee OA was considered present if having ICRS Grade III on one surface or Grade II on a minimum of two opposing cartilage surfaces in the compartment. Clearly localised traumatic cartilage damage was not considered as OA, irrespective of depth. The knee was defined as ACL‐stable if all ACL stability tests were negative = 0 (Lachman, Forward Drawer and Pivot‐shift tests).

### Surgical technique

The arthroscopies were performed by a wide variety of surgeons, from residents and trauma‐specialists to specialists in knee arthroscopy, where the latter performed 87% of all surgeries and 96% of repairs. In most cases of meniscal repair, the surfaces of the meniscal tears were abraded with a diamond‐coated rasp whereafter the meniscus was sutured. Typically, tears in the posterior horn and body were sutured with all‐inside Fast‐Fix anchors or in a few rare cases during 2010, meniscal arrows. Tears in the anterior part were sutured with an inside‐out or an outside‐in technique. Sometimes small holes were made in the femoral notch with a Steadman pick, to induce bleeding with bone marrow into the joint. All ACLR were given pre‐op antibiotics. Hamstrings grafts were used in almost all cases of ACLR.

### Statistical analysis

The following analyses were performed: (a) estimating of incidence rates of reoperations and complications in the whole cohort; (b) comparison of reoperations rates between males and females in the whole cohort; and (c) comparison of reoperations rates between partial meniscectomy and meniscal repair in the subgroup 15–40 years. Further, reoperations rates between partial meniscectomy and meniscal repair were compared stratified by ACL status and, lastly, stratified by compartment (medial, lateral or both menisci). Patients were followed until end of study period 2019‐12‐31 or death. Time to the first reoperation during follow‐up is presented using Kaplan‐Meier survival curves. Rates of reoperations in the total cohort and the subgroup were estimated in two ways. In the analysis of first reoperations (any reoperation or reoperations in the same meniscus), each patient was followed‐up from index surgery to first reoperation, death or 2019‐12‐31. In analysis of all reoperations, an Andersen‐Gill model was assumed, where each person contributes with all reoperations observed and the time until end of study or death. Incidence rates are reported per 1000 person‐years with 95% jackknife confidence intervals derived with function *stptime* in Stata.

Rates of reoperations were compared between groups, males and females in the whole cohort, or patients undergoing partial meniscectomy versus meniscal repair in the subgroup, using flexible parametric survival models (with 2 degrees of freedom) with first reoperation as outcome. The models were adjusted for pre‐specified confounders: age, sex (in analysis of meniscal repair vs. partial meniscectomy), surgery type (meniscal repair vs. partial meniscectomy, in the analysis of sex), tear type, knee laterality, body mass, inverse of height squared, smoking, operated menisci (medial, lateral, both) and ACL status. To estimate if ACL status at the index surgery modifies the association between meniscal repair/partial meniscectomy and reoperations, an interaction was included between surgery type (meniscal repair vs partial meniscectomy) and ACL status at index surgery. Relative risks (hazard ratio [HR]) as well as difference in absolute risk are reported. The number of missing values in relevant variables are presented in table footnotes and was low. Persons with missing data in relevant variables were excluded from the regression models. Due to the epidemiological nature of the study, no corrections for multiple testing were used. All estimates are reported with 95% confidence intervals (CI). Data were entered in a secure Excel spreadsheet (Microsoft® Corp., Redmond, WA). IBM© SPSS© Statistics, version (29.0.0.0) and Stata 18 were used for the statistical analyses.

## RESULTS

Preoperative patient characteristics are presented in Table 1 and operative findings in Table 2.

**Table 2:** Surgical characteristics of the whole study cohort.

	All patients
	*N* = 2098
Type of surgery	
Partial meniscectomy	1703 (81.2)
Meniscus repair	395 (18.8)
ACL visual status	
Bleeding	6 (0.3)
Normal	1217 (58.0)
Partial tear	153 (7.3)
Previous ACLR	88 (4.2)
Acute rupture	42 (2.0)
Old rupture	590 (28.1)
ACL stability	
Intraoperatively stable knee	1314 (62.6)
Intraoperatively unstable knee	362 (17.3)
ACLR at Index surgery	422 (20.1)
Antibiotics^a^	455 (24.4)
Tear type	
Bucket‐handle	650 (31.0)
Degenerative	608 (29.0)
Flap	469 (22.4)
Horizontal	54 (2.6)
Complex	7 (0.3)
Longitudinal	194 (9.2)
Radial	116 (5.5)
Discoid meniscus	35 (1.7)
Medial tear	1623 (77.4)
Lateral tear	767 (36.6)
Medial suture	280 (13.3)
Lateral suture	135 (6.4)

*Note*: Numbers are *n* (%).

Abbreviations: ACL, anterior cruciate ligament; ACLR, ACL reconstruction; APM, arthroscopic partial meniscectomy.

^a^ Missing data: antibiotics *n* = 233 (11.1%).

### Reoperations

**Figure 3:**
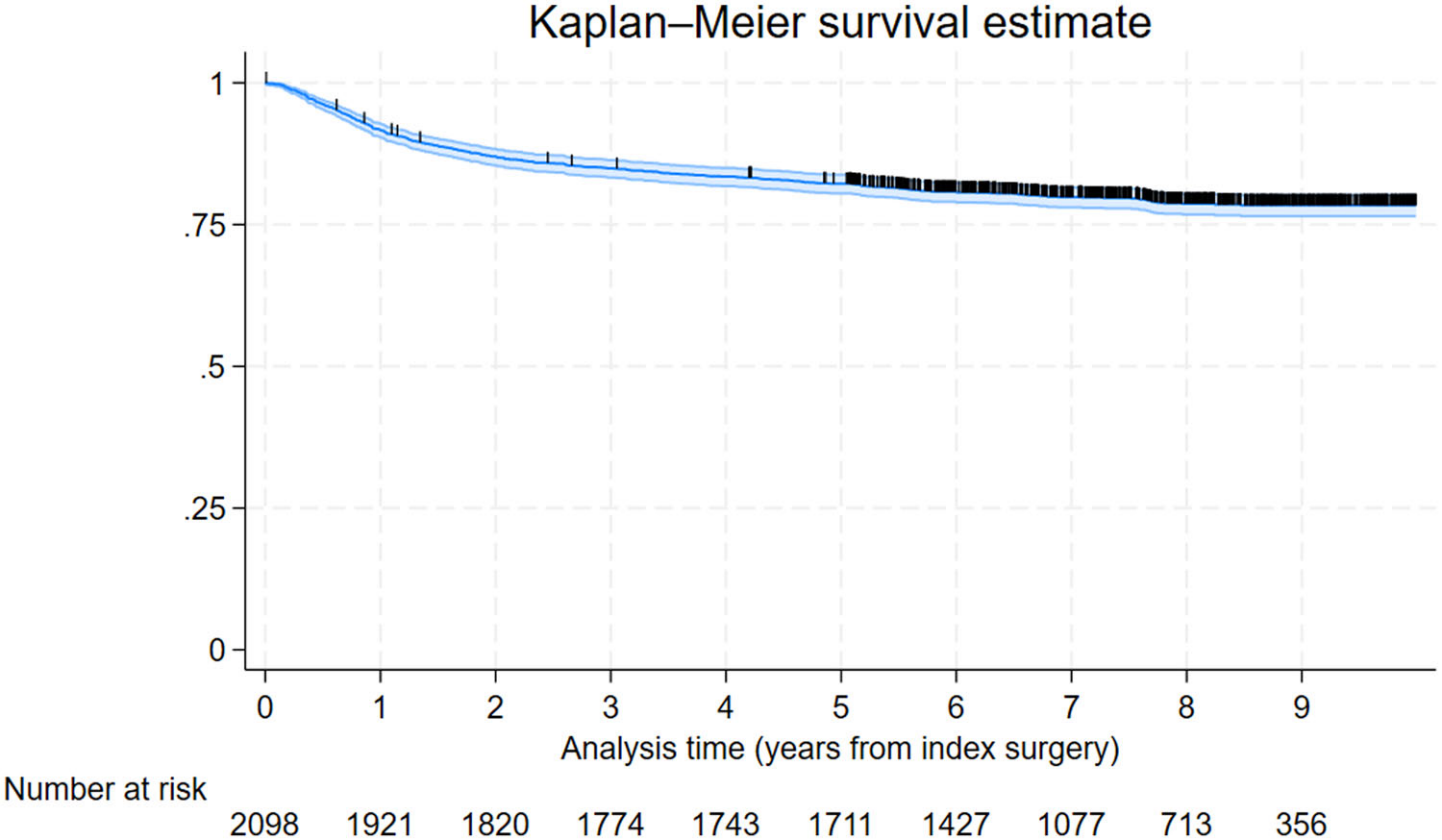
Kaplan–Meier graph showing time to first knee reoperation for all causes in the whole cohort. The blue area shows 95% confidence interval.

Study participants had a total follow‐up time of 13,624 person‐years. The incidence rate of the first reoperation was 32 per 1000 person‐years (95% CI 29–35). Figure 3 shows time to first reoperation. Considering all reoperations (i.e. allowing for multiple reoperations), the study had 16,081 person‐years of observation time with 540 reoperations in total, which leads to a reoperation rate of 34 (95% CI 31–37) per 1000 person‐years. Reoperations details in Table 3.

**Table 3:** Reoperations and complications in the whole study cohort.

	All patients
Number of patients with reoperations, *N* (%)	
1 Reoperation	337 (16.1)
2 Reoperations	80 (3.8)
3 Reoperations	9 (0.4)
4 Reoperations	4 (0.2)
Total number of reoperations, *N*	540
Total number of reoperated patients, *N* (%)	430 (20.5)
Number of complications, *N* (%)	47 (2.1)
Number of complications in patients without concomitant ACLR, *N* (%)	25 (1.5)

Abbreviation: ACLR, anterior cruciate ligament reconstruction.

The reoperation rate (including TKA) of the same meniscus was 19 (17–21) per 1000 person‐years.

In both the whole cohort and in the subgroup, the most commonly performed surgery at the first reoperation was a new meniscus surgery. Complete list of reoperations in Supporting Information: Appendix Table 11.

Number of later ACLRs performed within 4 months of index surgery in the whole cohort was 26 patients (27.4%) out of 95 ACLR performed as first reoperation.

### Postoperative complications

Proportion (95% CI) of postoperative complications was 2.1% (1.6–2.9) within 2 years from index surgery (29 patients in partial meniscectomy group and 16 in meniscal repair group, described in Table 4). They occurred with a median of 26 days (range: 6–135) from index surgery.

**Table 4:** List of surgery complications in all 2098 patients (47 complications in 45 patients).

*N* = 47	Complications (45 patients)	Mean age	Mean BMI	Women, *N*	Meniscus repair, *N*	APM, *N*
1	Acute compartment syndrome	34	41.3	0	0	1
13	Arthrofibrosis	36.2	26.5	4	7	6
2	Inflammatory arthritis	36.5	27.8	2	0	2
5	Major knee bleeding	33.8	23.3	0	1	4
4	Nerve injury/pain	29.3	23.8	1	3	1
4	Other	53.8	21.9	2	0	4
6	Septic arthritis	33.3	24.6	1	2	4
6	Superficial wound infection	28.8	23.8	1	3	3
6	VTE/pulmonary embolism (1)	47.5	26.4	3	0	6

*Note*: Complete list in Supporting Information: Appendix Table 9.

Abbreviations: APM, arthroscopic partial meniscectomy; BMI, body mass index; VTE, venous thrombus embolism.

### Sex differences

Males had lower rates of reoperation than females, crude HR 0.65 (95% CI 0.56–0.82) and fully adjusted HR of 0.61 (95% CI 0.47–0.80). This corresponded to 8.5% less reoperations in males 5 years after index surgery (95% CI −13.4 to −3.6). (Details in Supporting Information: Appendix S1).

### Comparison of meniscal repair versus partial meniscectomy in subgroup 15–40 years old

Table 1 presents subgroup patient characteristics and operative findings are presented in Table 5.

**Table 5:** Surgical data for the subgroup 15–40 years, only bucket‐handle, longitudinal and horizontal meniscus tears.

	All	APM	Meniscus repair
*N*	702	361	341
Torniquet	701 (99.9)	360 (99.7)	341 (100)
Antibiotics^a^	307 (46.9)	132 (39.9)	175 (54.2)
ACL visual status			
Bleeding	3 (0.4)	0 (0)	3 (0.9)
Normal	214 (30.4)	124 (34.3)	90 (26.4)
Partial tear	49 (7.0)	27 (7.5)	22 (6.5)
Previous ACLR	41 (5.8)	22 (6.1)	19 (5.6)
Acute rupture	28 (4.0)	7 (1.9)	21 (6.2)
Old rupture	367 (52.3)	181 (50.1)	186 (54.5)
ACL stability			
Intraoperatively stable knee	253 (36.0)	143 (39.6)	110 (32.3)
Intraoperatively unstable knee	157 (22.4)	99 (27.4)	58 (17.0)
ACLR at Index surgery	292 (41.6)	119 (33.0)	173 (50.7)
Tear type			
Bucket‐handle	509 (72.5)	300 (83.1)	209 (61.3)
Displaced bucket‐handle	253 (36.0)	151 (41.8)	102 (29.9)
Horizontal	33 (4.7)	25 (6.9)	8 (2.3)
Longitudinal	160 (22.8)	36 (10.0)	124 (36.4)
Medial tear	536 (76.4)	265 (73.4)	271 (79.5)
Lateral tear	289 (41.2)	140 (38.8)	149 (43.7)
Medial suture	255 (36.3)	n/a	255 (74.8)
Lateral suture	106 (15.1)	n/a	106 (31.1)

*Note*: Numbers are *n* (%).

Abbreviations: ACL, anterior cruciate ligament; ACLR, anterior cruciate ligament reconstruction; APM, arthroscopic partial meniscectomy.

^a^ Missing data: antibiotics: APM *n* = 30 (8.3%) meniscus repair *n* = 18 (5.3%).

**Figure 4:**
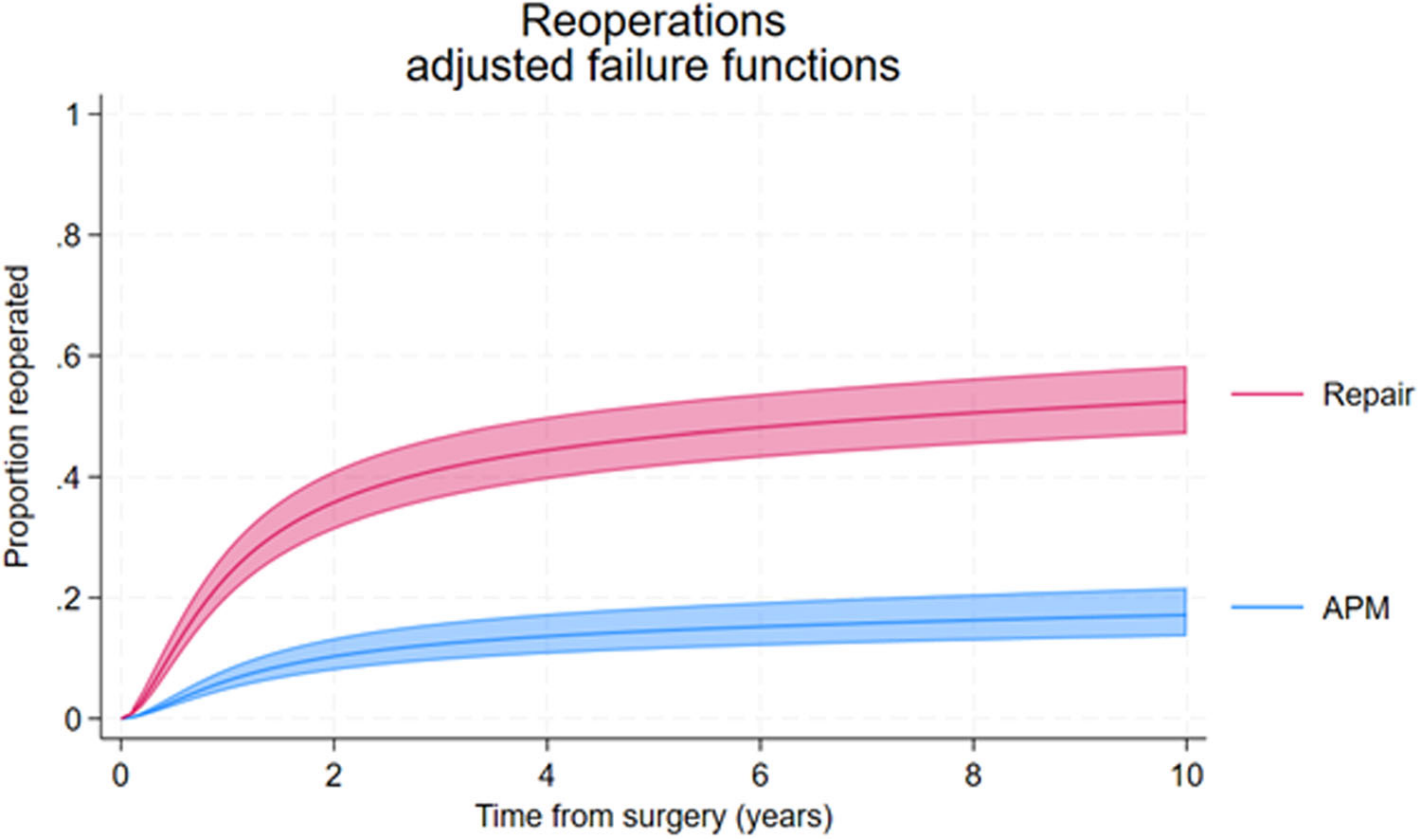
The (adjusted) proportion (95% confidence interval [CI]) reoperated for all causes in subgroup 15–40 years old. Coloured areas show 95% CIs.

Reoperation for all causes was more than four times more common in the meniscus repair group versus partial meniscectomy, the unadjusted HR was 3.8 (95%CI 2.8–5.1) and when adjusting for confounders it was 4.3 (95%CI 3.1–6.0). Number of reoperations described in Table 6. Five years after index surgery, 46% (95%CI 42–52) of the repairs were reoperated, while only 15% (95%CI 11–18) in the partial meniscectomy group (Figure 4). The meniscus repair reoperation incidence rate was 105 (95%CI 90–122) per 1000 person‐years, compared to in the partial meniscectomy group with an incidence rate of 24 (95%CI 18–31) per 1000 person‐years.

**Table 6:** Reoperations and complications in subgroup 15–40 years old.

	All	APM	Meniscus repair
Number of patients with reoperation			
1 Reoperation	167 (23.8)	48 (13.3)	119 (34.9)
2 Reoperations	52 (7.4)	9 (2.5)	43 (12.6)
3 Reoperations	2 (0.3)	1 (0.3)	1 (0.3)
4 Reoperations	1 (0.1)	0	1 (0.3)
Total number of reoperations, *N*	281	69	212
Number of reoperated patients	222 (31.6)	58 (16.1)	164 (48.1)
Number of other complications	23 (3.3)	8 (2.2)	15 (4.4)
Number of complications in patients without concomitant ACLR	7 (1.0)	2 (0.6)	5 (1.5)

*Note*: Numbers are *n* (%).

Abbreviations: ACLR, anterior cruciate ligament reconstruction; APM, arthroscopic partial meniscectomy.

**Figure 5:**
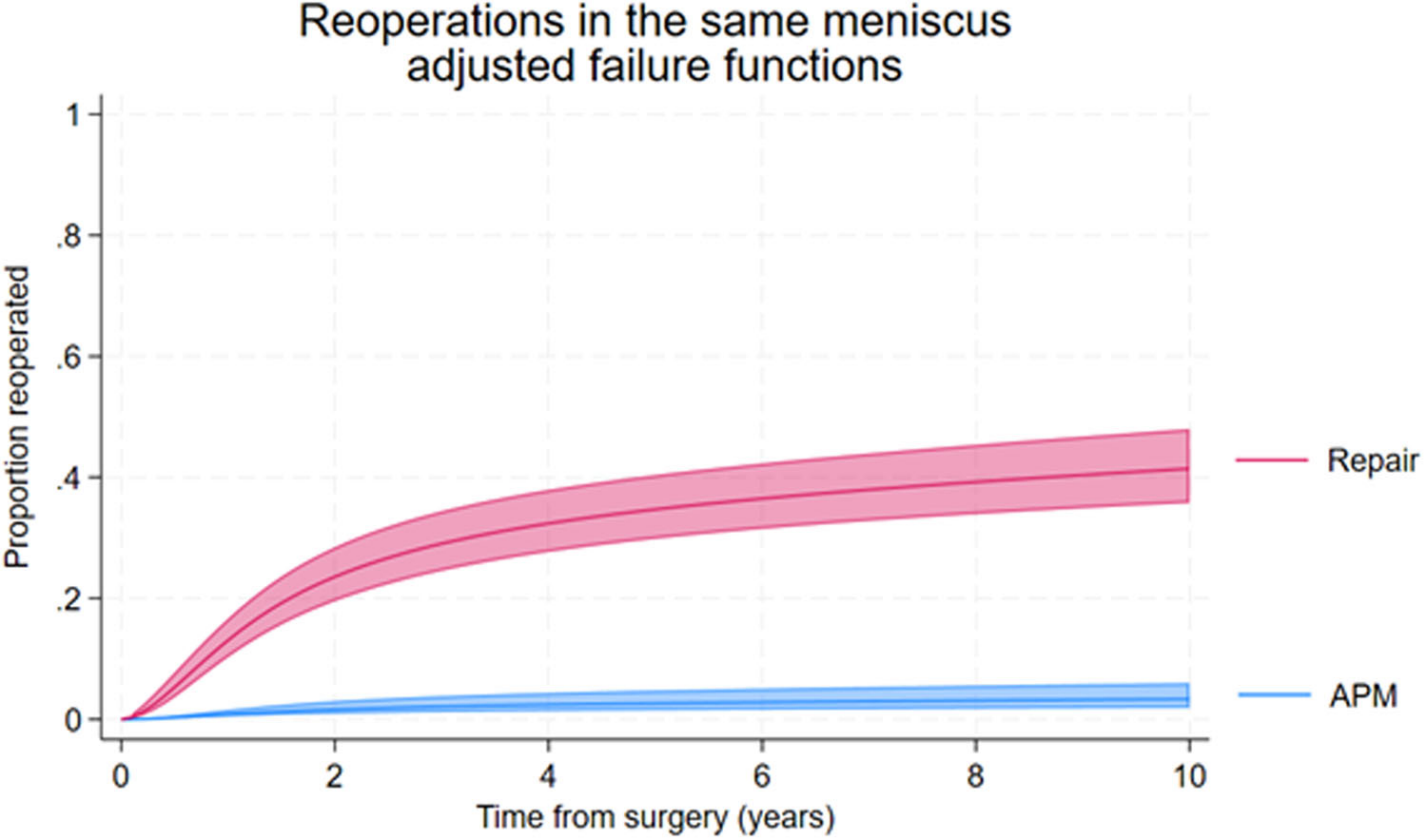
The (adjusted) proportion reoperated in the same meniscus, group 15–40 years. Coloured areas show 95% confidence intervals.

Among meniscal repairs the rate of reoperations in the same meniscus was increased 17‐fold (95%CI 9–31) compared to partial meniscectomy when adjusted for confounders (crude HR for reoperation was 15 [95% CI 8–28]). At 5 years after index surgery, 34.7% (95%CI 29.8–40.4) had been reoperated on in the meniscal repair group while only 2.6% (95%CI 1.5–4.8) in the partial meniscectomy group (Figure 5). In the meniscal repair group, the rate of reoperation in the same meniscus was 68.3 (95% CI 56.6–82.8) per 1000 person‐years (126 reoperations in 341 persons) and in the partial meniscectomy group 4.0 (95% CI 2.2–7.8) per 1000 person‐years (11 reoperations in 361 persons).

### Postoperative complications in the subgroup 15–40 years old

There were 3.3% (95% CI 2.1–4.8) postoperative complications within 2 years from index surgery (15 patients with meniscal repair and 8 partial meniscectomy), listed in Table 7. They occurred with a mean 57 days (SD 14.9) and median of 30.0 days (SD 71.2, range 1–286) from index surgery.

**Table 7:** List of surgical complications, subgroup 15–40 years old, with bucket‐handle, longitudinal or horizontal meniscal tear.

*N* = 24	Complications (23 patients)	Mean age	Mean BMI	Women, *N*	Meniscus repair, *N*	APM, *N*
9	Arthrofibrosis	31.3	26.3	3	6	3
4	Major knee bleeding	29.3	22.5	0	1	3
3	Nerve injury/pain	23.3	22.9	1	3	0
2	Septic arthritis	21.5	21.9	0	2	0
5	Superficial wound infection	25.2	23.44	1	3	2
1	Venous thrombus embolism	33	26.1	0	0	1

*Note*: 24 complications in 23 patients. Complete list in Supporting Information: Appendix Table 10.

Abbreviations: APM, arthroscopic partial meniscectomy; BMI, body mass index.

### ACL‐status and risk of reoperations in subgroup 15–40 years old

Reoperation was more common among patients with isolated meniscus repair, 89 out of 168 (53%) than in meniscus repair patients with concomitant ACLR, 75 out of 173 (43%), Table 8.

**Table 8:** Risk of reoperation dependant on ACL stability, subgroup 15–40 years old, with bucket‐handle, longitudinal and horizontal tears.

	Incidence rate per 1000 person‐years (95% CI) meniscus repair APM		Adjusted HR meniscus repair vs. APM (95% CI)	Meniscus repair, *N*	APM, *N*
Reoperation, all causes					
Stable	88 (67–117)	8 (4–16)	8 (4–17)	110	143
Unstable	246 (181–336)	35 (23–54)	5 (3–9)	58	99
ACLR at index	88 (71–111)	36 (25–52)	2 (1–4)	173	119
Reoperation in the same meniscus					
Stable	82 (62–109)			110	
Unstable	82 (54–123)			58	
ACLR at index	57 (44–74)			173	

Abbreviations: ACL, anterior cruciate ligament; ACLR, anterior cruciate ligament reconstruction; APM, arthroscopic meniscus repair.

**Figure 6:**
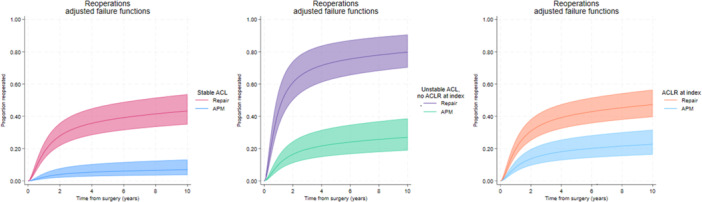
The (adjusted) proportion reoperated for all causes in group: stable, unstable and anterior cruciate ligament reconstruction (ACLR). Coloured areas show 95% confidence intervals.

The reoperations rates for all causes after partial meniscectomy versus repair differed depending on the ACL status, Figure 6.

**Figure 7:**
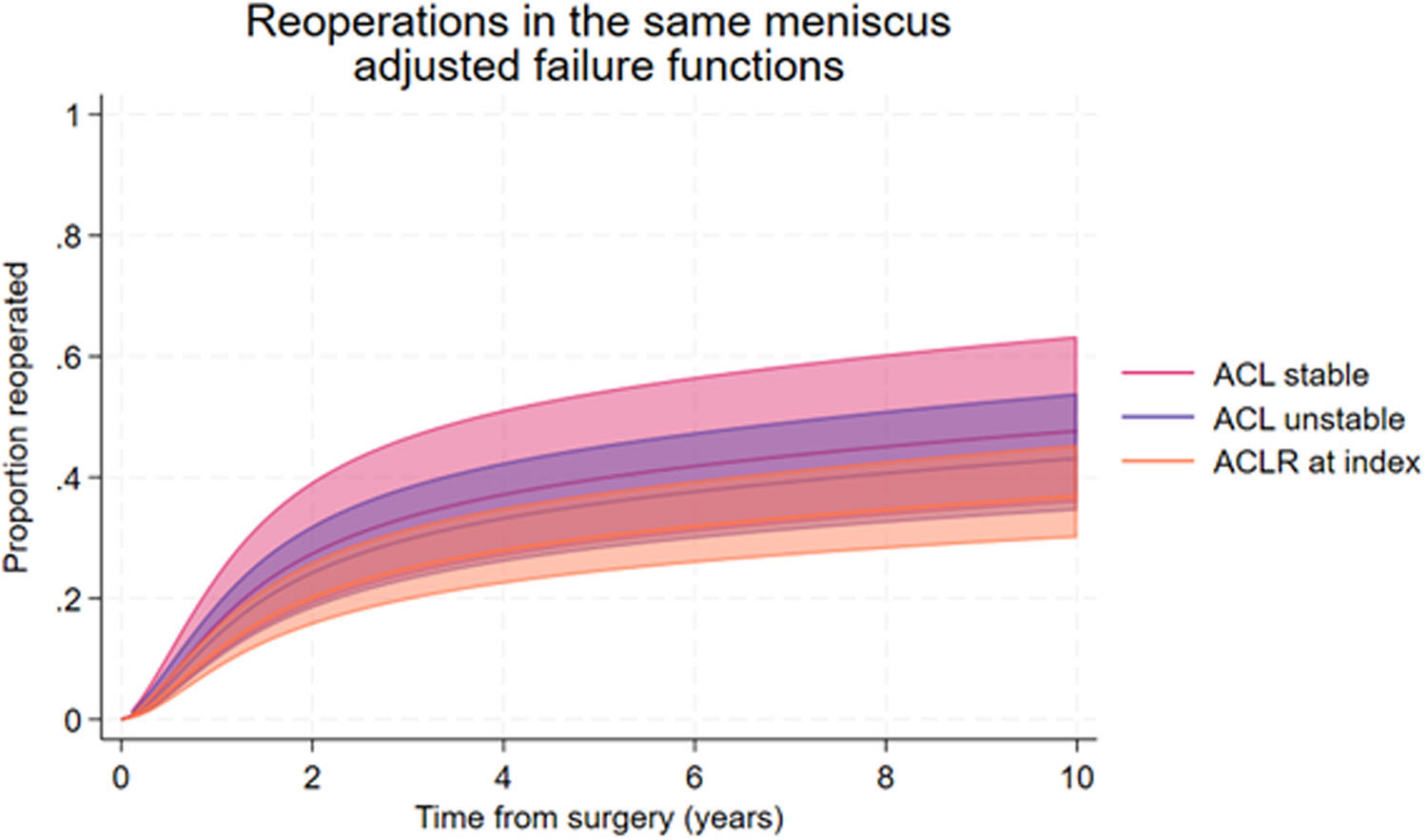
Knee stability groups, adjusted cumulative incidence curves, same meniscus reoperations. Coloured areas show 95% confidence intervals.

The effect of knee stability on reoperation rate in the same meniscus was only analysed in meniscus repair group, due to small numbers in the partial meniscectomy group. There was no evidence of differences in reoperations in the same meniscus based on ACL status but the uncertainty around the estimates was considerable. Reoperation in the same meniscus could be more common in stable knees compared to knees with ACLR at index, adjusted HR (95% CI) 1.24 (0.81–1.90). Comparing unstable knees versus ACLR at index, reoperation in the same meniscus could be more common in unstable knees, HR 1.44 (0.87–2.38), Figure 7.

In meniscus repair patients with a complete ACL tear that did not have concomitant ACL surgery, the number of ACLRs performed within 4 months of index surgery in the subgroup 15–40 was 15 patients (50%) out of 30 ACLR after index.

### Medial versus lateral

Comparing reoperation for any cause of the medial and lateral meniscus, the difference in proportion reoperated at 5 years, between repair and partial meniscectomy was 37% (95%CI 29%–45%) in medial meniscus, 23% (95%CI 11%–36%) in lateral meniscus and 26% (95%CI 10%–41%) when both menisci were operated on.

Medial meniscus repairs had a 53% higher risk than lateral repairs of a reoperation *in the same meniscus*, HR 1.53 (95%CI 0.97–2.43). Incidence rate of reoperation in the same meniscus was 80/1000 person years (95%CI 63–101) in the medial meniscus, 57/1000 person years (95%CI 38–88) in the lateral meniscus and 46/1000 person years (95%CI 28–81) if both menisci were repaired.

### Suture material

The study was not intended to compare suture materials or techniques. Of the 395 meniscal repair surgeries, Fast‐Fix suture anchors were used in 359, whereof a combination of Fast‐Fix and inside‐out or outside‐in sutures were used in 21 cases. The study also included 15 patients with meniscal arrows, with 10 patients reoperated, eight because of suture failure.

## DISCUSSION

The main findings of the study were that one in five patients with meniscus surgery had any ipsilateral knee reoperation, with more than half of these being within 2 years of the index surgery. Among patients below 40 years of age, there was a four‐fold increase in reoperation of all causes and a 17‐fold increase in same meniscus reoperations after meniscal repair compared with partial meniscectomy.

In counselling patients about meniscus injury treatment, it is important to have knowledge of potential differences in terms of risk of complications and reoperations, as well as expected suture survival rate. Highly cited studies on partial meniscectomy versus meniscal repair by Paxton et al. and Stein et al. are now more than 13 years old and since arthroscopic surgery continues to evolve, new updated data is important [25, 37]. In a register study of meniscal repair by Lyman et al. on 9605 American patients, the rate of failure, measured as reoperation with partial meniscectomy after meniscal repair was 8.9% for 3 years mean follow‐up, with a markedly better performance for high volume surgeons [16]. The authors did not report risk for other reoperations than partial meniscectomy. The present study differs both in length of follow‐up as well as a more comprehensive description and inclusion of all reoperations performed. High volume surgeons have been suggested to have lower reoperation rates after meniscus repair, and though most repairs in our study were performed by experienced surgeons, a number of the less complicated surgeries were delegated to surgeons with few meniscus surgeries per year. There are huge differences both worldwide and even within the same country, according to how, why and how frequently partial meniscectomies and meniscal repairs are performed. When comparing studies from different countries, one important factor is that different systems for delivering healthcare influence the frequency of arthroscopic procedures [9].

In this study, the whole cohort and especially the subgroup aged 15–40 years displayed high proportions of potential seriously injured knees with 72% bucket‐handle meniscus tears and 64% ACL injuries. The somewhat higher numbers of reoperations in this study cohort compared with other studies could be explained by the presence of a high proportion of severely injured knees with worse prognosis. Also, the finding that more bucket‐handle meniscal tears had partial meniscectomy than a meniscal repair, even in the subgroup aged 15–40 years suggests a potential underutilization of attempts at meniscal repair. It has been reported that ACL‐injured patients have more meniscal damage with longer time between injury and surgery [5, 19].

Previous research has suggested that meniscus repairs concomitant with ACLR results in better meniscus healing rates, medial meniscus repairs have higher reoperation‐rates than lateral repairs, and intrameniscal arrow implants are inferior to all‐inside sutures [7, 13, 27, 29, 32]. This study also confirmed all these findings. The patients with concomitant ACLR did have both fewer all cause reoperations and same meniscus reoperations than meniscal repair alone in unstable knees.

The finding that more men receive meniscus surgery, but women have almost 30% higher both all causes reoperation rates and same meniscus reoperations is somewhat difficult to explain. Studies report that women tend to have a longer recovery time and lower KOOS scores after a meniscus injury, which may influence the risk of reoperation [17]. One explanation could be that men are more willing to choose primary surgery while women included for meniscus surgery have more severe knee injuries or that women more often report post‐operative symptoms leading to a reoperation [2].

The low number of complications, despite including also minor complications not coded in registries, ranging between 1.5% for partial meniscectomy alone to 4.4% for meniscal repair with concomitant ACLR, is in line with the literature [14, 36]. This supports the notion that arthroscopic meniscus surgery is a safe procedure in a routine healthcare setting. Moreover, the most frequent complication was arthrofibrosis which might as well be a consequence of the initial knee trauma and not necessarily due to surgery. The number of postoperative infections was very small, and though not the aim to study this, the study supports not routinely giving preoperative antibiotics in meniscus surgery without ACLR.

A strength of the study is the large numbers of included patients undergoing meniscus surgery and studied in detail in a routine healthcare setting with a long‐time follow‐up. The use of prospectively ascertained data from included patients and surgical charts from the included hospitals contributes to high external validity. The study depicts patients operated on 'in real life', without the limiting patient selections commonly present in trials when study results usually are not generalisable for the whole population and a variable level of experience among surgeons. Further, the study incorporates data on BMI, smoking, OA, ACL status and any concomitant knee injuries, reoperations and complications from our medical records which is more accurate than studies relying on patient self‐report [34].

This study had some limitations. With the numerous surgeons involved, there are potential variability in surgeon‐reported knee OA and meniscal injury data. A major limitation is the lack of information on activity level and subjective knee symptoms for all patients both before and after surgery, as this could differ between groups. Another study on 421 patients from the same cohort has suggested that KOOS‐scores at mean 4 years postoperatively is almost the same for both partial meniscectomy and meniscal repair [2]. Interestingly, the KOOS for patients with concurrent ACLR are higher than after meniscus surgery alone, irrespective of patients age [2].

Importantly, the observational nature of the data, as arthroscopic surgeries were not randomly assigned to subjects, creates confounding by indication (i.e., there is a patient selection by the surgeon which may influence risk of reoperation and complications). Further, the data lack date of meniscus injury, therefore time from injury to surgery can't be measured. In a study from Ronnblad et al., this was however suggested not to essentially influence the healing rate [29]. There may also be missing some reoperations done outside the Scania region, or by private care, but this ought to be minimal within the follow‐up period.

## CONCLUSIONS

In conclusion, the present study found a four‐fold increased rate of reoperation after meniscal repair vs partial meniscectomy, higher risk after medial vs lateral meniscus repairs, but a low rate of other medical complications in a routine healthcare setting. The information should be considered together with all other considerations by the surgeon and the patient when counselling before meniscus surgery.

## AUTHOR CONTRIBUTIONS

Fredrik Boric‐Persson (responsible) was involved in study conception and design, data collection, the data analysis, interpretation of results, and drafted the manuscript. Aleksandra Turkiewicz (statistician) was involved in study design, data analysis and manuscript draft editing. Martin Englund (supervisor) was involved in manuscript draft editing. Paul Neuman (lead investigator) was involved in study conception and design, interpretation of results, and manuscript drafting and editing. All authors read and approved the final manuscript.

## CONFLICT OF INTEREST STATEMENT

Martin Englund reports consultancy for Grünenthal Sweden and Key2Compliance. Paul Neuman: Consultant fees has been received infrequently from Arthrex and Smith & Nephew during medical education events and instructional courses. The other authors declare no conflicts of interest.

## ETHICS STATEMENT

The study was approved by the Regional Lund University Ethics Committee (IRB 2016‐873/2017‐02‐07 and IRB 2019‐02850/20191121) and it adheres to the rules of the Helsinki Declaration of 1975, as revised in 2000. Patients were publicly informed about the study and the option of opting out of the study publication in concordance with the ethical approval.

## Supporting information

KSSTA_ReopCompl_10y_MR_APM_APPENDIX_revMINOR_prod.

## Data Availability

Author elects to not share dataResearch data are not shared. The individual data are neither publicly available nor available for data sharing due to ethical restrictions and national legislation of use of Swedish healthcare data.
