# Supplementary material for: Increased reoperation rates after meniscus repair compared to arthroscopic partial meniscectomy: Data from a comprehensive clinical cohort with up to 10 years follow‐up
Source: Knee Surg Sports Traumatol Arthrosc. 2025 Jul 21;34(6):1946–59. doi: 10.1002/ksa.12791 (PMC13266913; doi:10.1002/ksa.12791)
Supplement: Supplementary file 1 — KSSTA_ReopCompl_10y_MR_APM_APPENDIX_revMINOR_prod. [file KSA-34-1946-s001.docx]

**APPENDIX**

Increased reoperation rates after meniscus repair compared to arthroscopic partial meniscectomy - data from a comprehensive clinical cohort with up to 10 years follow-up.

**Further analysis of gender differences**

**The whole cohort**

Reoperation percentage for all causes was 25.4% (178 patients) for women and 18.0% (252 patients) for men with a difference of 7.4% (95%CI 3.7-11.0). The percentage of new surgery to the same meniscus was 18.4% (129 patients) for women and 13.0% for men (144 patients) with a difference of 8.1% (95CI 5.1, 11.1).

Reoperation percentage after partial meniscectomy for any cause was 18.6% for women and 13.1% for men, with a difference of 5.5% (95%CI 1.9, 9.1). For reoperation in the same meniscus after partial meniscectomy it was 12.7% for women and 6.0% for men with a difference of 6.8% (95%CI 4.0, 9.5).

For meniscal repair the percentage of reoperation for all causes was 50.0% (76 patients) for women and 41.6% (101 patients) for men with a difference of 8.4% (95%CI -1.7,18.5). The percentage of new surgeries to the same meniscus after meniscal repair was 38.8% (59 patients) for women and 30.9% (75 patients) for men with a difference of 8.0% (95%CI -1.7, 17.6).

**The subgroup 15-40 years**

Reoperation percentage after partial meniscectomy for any cause was 16.7% (15 patients) for women and 15.9% (43 patients) for men with a difference of 0.8% (95%CI -8.0, 9.8). Reoperation in the same meniscus after partial meniscectomy was 3.3% (3 patients) for women and 3.0% (8 patients) for men with a difference of 0.4% (95%CI -3.7, 4.5).

Reoperation percentage for all causes after meniscal repair was 51.9% (69 patients) for women and 45.7% (95 patients) for men with a difference of 5.6% (95%CI -4.7,17.1).

Reoperation percentage of new surgeries to the same meniscus after meniscal repair was 40.6% (54 patients) for women and 34.6% (72 patients) for men with a difference of 5.4% (95%CI -4.6, 16.5).

**Table 9:** Detailed list of postoperative complications in all 2098 patients. (47 complications in 45 patients)

| N | Repair | APM | ACLR | Complication, type | Age | BMI | Sex | Smoker | Comment |
| --- | --- | --- | --- | --- | --- | --- | --- | --- | --- |
| 1 |  | yes |  | Acute compartment syndrome | 34 | 41,3 | M |  |  |
| 2 | yes |  | yes | Arthrofibrosis | 37 | 29,0 | M |  |  |
| 3 |  | yes | yes | Arthrofibrosis | 23 | 21,4 | M |  |  |
| 4 | yes |  |  | Arthrofibrosis | 31 | 27,8 | M |  |  |
| 5 |  | yes |  | Arthrofibrosis | 42 | 26,5 | F |  |  |
| 6 |  | yes | yes | Arthrofibrosis | 23 | 23,9 | M |  |  |
| 7 |  | yes | yes | Arthrofibrosis | 44 | 27,1 | M |  |  |
| 8 |  | yes | yes | Arthrofibrosis | 37 | 29,4 | M | S |  |
| 9 | yes |  |  | Arthrofibrosis | 79 | 29,6 | M |  |  |
| 10 | yes |  | yes | Arthrofibrosis | 23 | 24,7 | F |  |  |
| 11 | yes |  | yes | Arthrofibrosis | 35 | 24,6 | M |  |  |
| 12 | yes |  | yes | Arthrofibrosis | 40 | 25,1 | F |  |  |
| 13 | yes |  |  | Arthrofibrosis | 22 | 28,1 | F |  |  |
| 14 |  | yes | yes | Arthrofibrosis | 34 | 27,0 | M | S |  |
| 15 | yes |  | yes | Chronic compartment syndrome | 23 | 24,5 | M |  |  |
| 16 |  | yes |  | Death 2 days postoperatively | 54 | 22,2 | M | S | Cause unknown |
| 17 |  | yes |  | Inflammatory arthritis | 42 | 23,0 | F |  |  |
| 18 |  | yes |  | Inflammatory arthritis | 31 | 32,5 | F | S |  |
| 19 |  | yes | yes | Major knee bleeding | 40 | 22,0 | M |  |  |
| 20 |  | yes | yes | Major knee bleeding | 34 | 27,0 | M | S |  |
| 21 | yes |  | yes | Major knee bleeding | 19 | 18,6 | M |  |  |
| 22 |  | yes |  | Major knee bleeding | 24 | 22.3 | M |  |  |
| 23 |  | yes |  | Major knee bleeding | 52 | 26,7 | M |  | Mb.VonWillebrand |
| 24 | yes |  |  | Nerve injury | 22 | 20.3 | F |  |  |
| 25 | yes |  |  | Neuroma | 25 | 24,0 | M | S | Reoperation |
| 26 |  | yes |  | Osteonecrosis in knee | 77 | 24,5 | F |  |  |
| 27 |  | yes |  | Patellar tendinitis | 48 | 20,7 | M | S |  |
| 28 |  | yes |  | Portal ganglion | 36 | 20,1 | F |  |  |
| 29 |  | yes |  | Postoperative chronic pain | 47 | 26,4 | M |  |  |
| 30 |  | yes | yes | Pulmonary embolism | 43 | 27,4 | M |  |  |
| 31 | yes |  |  | Septic arthritis | 19 | 19.5 | M | S |  |
| 32 |  | yes | yes | Septic arthritis | 31 | 29,0 | M |  |  |
| 33 |  | yes |  | Septic arthritis | 52 | 27,8 | M |  |  |
| 34 |  | yes | yes | Septic arthritis | 22 | 22,9 | M | S |  |
| 35 |  | yes |  | Septic arthritis | 52 | 23,8 | F |  |  |
| 36 | yes |  | yes | Septic arthritis | 24 | 24,3 | M |  |  |
| 37 |  | yes |  | Superficial wound infection | 47 | 25,3 | M |  |  |
| 38 |  | yes |  | Superficial wound infection | 28 | 22.8 | M |  |  |
| 39 |  | yes | yes | Superficial wound infection | 29 | 25,5 | M |  |  |
| 40 | yes |  | yes | Superficial wound infection | 16 | 18,0 | F |  |  |
| 41 | yes |  | yes | Superficial wound infection | 27 | 23,1 | M |  |  |
| 42 | yes |  | yes | Superficial wound infection | 26 | 27,8 | M |  |  |
| 43 |  | yes |  | VTE | 62 | 23,8 | F |  |  |
| 44 |  | yes | yes | VTE | 33 | 26,1 | M |  |  |
| 45 |  | yes |  | VTE | 45 | 28,7 | M |  |  |
| 46 |  | yes |  | VTE | 50 | 28,3 | F |  |  |
| 47 |  | yes |  | VTE | 52 | 23,8 | F |  |  |
|  |  |  |  |  |  |  |  |  |  |
| Total | 16 | 31 | 22 |  |  |  | F=14 | N=9 |  |

[APM=arthroscopic partial meniscectomy, ACLR= Anterior Cruciate Ligament Reconstruction]

**Table 10:** Detailed list of postoperative complications, subgroup 15-40 years old, with bucket handle, Longitudinal and Horizontal meniscus tears (30 complications in 29 patients)

| N | Repair | APM | ACLR | Complication, type | Age | BMI | Sex | Smoker |
| --- | --- | --- | --- | --- | --- | --- | --- | --- |
| 1 |  | yes |  | Acute compartment Syndrome | 34 | 41,3 | M |  |
| 2 | yes |  | yes | Arthrofibrosis | 37 | 29,0 | M |  |
| 3 |  | yes | yes | Arthrofibrosis | 23 | 21,4 | M |  |
| 4 | yes |  |  | Arthrofibrosis | 31 | 27,8 | M |  |
| 5 |  | yes | yes | Arthrofibrosis | 23 | 23,9 | M |  |
| 6 |  | yes | yes | Arthrofibrosis | 37 | 29,4 | M | S |
| 7 | yes |  | yes | Arthrofibrosis | 23 | 24,7 | F |  |
| 8 | yes |  | yes | Arthrofibrosis | 35 | 24,6 | M |  |
| 9 | yes |  | yes | Arthrofibrosis | 40 | 25,1 | F |  |
| 10 | yes |  |  | Arthrofibrosis | 22 | 28,1 | F |  |
| 11 |  | yes | yes | Arthrofibrosis | 34 | 27,0 | M | S |
| 12 | yes |  | yes | Chronic compartment ?compartment syndrome | 23 | 24,5 | M |  |
| 13 |  | yes |  | Inflammatory Arthritis | 31 | 32,5 | F | S |
| 14 |  | yes | yes | Major knee bleeding | 40 | 22,0 | M |  |
| 15 |  | yes | yes | Major knee bleeding | 34 | 27,0 | M | S |
| 16 | yes |  | yes | Major knee bleeding | 19 | 18,6 | M |  |
| 17 |  | yes |  | Major knee bleeding | 24 | 22,3 | M |  |
| 18 | yes |  |  | Nerve injury | 22 | 20,3 | F |  |
| 19 | yes |  |  | Neuroma | 25 | 24,0 | M | S |
| 20 |  | yes |  | Portal ganglion | 36 | 20,1 | F |  |
| 21 | yes |  |  | Septic arthritis | 19 | 19.5 | M | S |
| 22 |  | yes | yes | Septic arthritis | 31 | 29,0 | M |  |
| 23 |  | yes | yes | Septic arthritis | 22 | 22,9 | M | S |
| 24 | yes |  | yes | Septic arthritis | 24 | 24,3 | M |  |
| 25 |  | yes |  | Superficial wound infection | 28 | 22,8 | M |  |
| 26 |  | yes | yes | Superficial wound infection | 29 | 25,5 | M |  |
| 27 | yes |  | yes | Superficial wound infection | 16 | 18,0 | F |  |
| 28 | yes |  | yes | Superficial wound infection | 27 | 23,1 | M |  |
| 29 | yes |  | yes | Superficial wound infection | 26 | 27,8 | M |  |
| 30 |  | yes | yes | VTE | 33 | 26,1 | M |  |
| sum | 15 | 15 | 20 |  |  |  | F=7 | 7 |

[APM=arthroscopic partial meniscectomy, ACLR= Anterior Cruciate Ligament Reconstruction]

All listed nerve injuries in Table 8 and 9 were permanent, i.e. transient sensory deficits that had resolved at follow-up visit were not recorded as complications.

**Causes of reoperation**

**The whole cohort**

The most commonly performed surgery at the first reoperation was a new meniscus surgery (188 patients), ACLR with new meniscus surgery (27 patients), ACLR without new meniscus surgery (68 patients), osteotomy (15 patients) or TKA for OA (76 patients), hardware extraction (36 patients) and other (20 patients). The majority of ACLR reoperations were planned secondary procedures to the index meniscus surgery.

**Table11**: Procedure at reoperation (number of procedures, more than 1 procedure possible at the same surgery)

|  | **APM all** | **Repair all** | **APM subgroup** | **Repair Subgroup** |
| --- | --- | --- | --- | --- |
| **First reoperation** |  |  |  |  |
| **Patients** | N=253 | N=177 | N=58 | N=164 |
| **APM** | 60 | 108 | 7 | 98 |
| **Repair** | 9 | 11 | 1 | 11 |
| **ACLR** | 46 | 22 | 24 | 21 |
| **ACLR + APM** | 7 | 12 | 3 | 12 |
| **ACLR + Repair** | 3 | 5 | 0 | 4 |
| **Hardware extraction** | 20 | 16 | 15 | 15 |
| **HTO** | 15 | 0 | 2 | 0 |
| **TKA** | 76 | 0 | 0 | 0 |
| **Other** | 14 | 6 | 4 | 5 |
| **Complication surgery** | 4 | 3 | 1 | 2 |
|  |  |  |  |  |
| **Second reoperation** |  |  |  |  |
| **APM** | 8 | 33 | 3 | 31 |
| **Repair** | 0 | 3 | 0 | 3 |
| **ACLR** | 4 | 1 | 3 | 1 |
| **ACLR + APM** | 3 | 1 | 0 | 1 |
| **ACLR + Repair** | 1 | 0 | 0 | 0 |
| **Hardware extraction** | 9 | 6 | 2 | 6 |
| **HTO** | 4 | 1 | 1 | 0 |
| **TKA** | 2 | 0 | 0 | 0 |
| **Other** | 7 | 5 | 2 | 3 |
| **Complication surgery** | 5 | 2 | 0 | 2 |
|  |  |  |  |  |
| **Third reoperation** |  |  |  |  |
| **APM** | 2 | 1 | 0 | 1 |
| **Repair** | 1 | 0 | 0 | 0 |
| **ACLR** | 0 | 0 | 0 | 0 |
| **ACLR + APM** | 0 | 0 | 0 | 0 |
| **ACLR + Repair** | 0 | 0 | 0 | 0 |
| **Hardware extraction** | 2 | 1 | 1 | 1 |
| **HTO** | 0 | 0 | 0 | 0 |
| **TKA** | 1 | 1 | 1 | 0 |
| **Other** | 1 | 1 | 0 | 0 |
| **Complication surgery** | 3 | 0 | 0 | 0 |

[APM=arthroscopic partial meniscectomy, ACLR= Anterior Cruciate Ligament Reconstruction, HTO= high tibia osteotomy, TKA= Total Knee Arthroplasty, Complication surgery= Lavage due to septic arthritis, surgery to alleviate arthrofibrosis, lower leg compartment release, etc.]
